# Effect of UV Radiation and Temperature on Radical Scavenging Activity of *Hippophaë rhamnoides* L. and *Vaccinium oxycoccos* L. Fruit Extracts

**DOI:** 10.3390/ijms25189810

**Published:** 2024-09-11

**Authors:** Monika Michalak, Barbara Pilawa, Paweł Ramos, Ryszard Glinka

**Affiliations:** 1 Department of Pharmaceutical Sciences, Jan Kochanowski University, 25-369 Kielce, Poland; 2 Department of Biophysics, Faculty of Pharmaceutical Sciences in Sosnowiec, Medical University of Silesia, Katowice, 41-200 Sosnowiec, Poland; bpilawa@sum.edu.pl (B.P.); pawelramos@sum.edu.pl (P.R.); 3 Higher School of Social Sciences in Lublin, 20-102 Lublin, Poland

**Keywords:** *Hippophaë rhamnoides*, *Vaccinium oxycoccos*, plant extract, free radicals, antioxidants, UV radiation, storage conditions, EPR spectroscopy

## Abstract

New active ingredients, including those of plant origin, which could protect the skin against various harmful factors, such as UV radiation and free radicals responsible for skin ageing, are still being sought. The present study was focused on the antioxidant activity of *Hippophaë rhamnoides* L. and *Vaccinium oxycoccos* L. fruit glycolic extracts. Investigations were also carried out to evaluate the effect of UVA radiation and the storage of the sea buckthorn and European cranberry extracts at an elevated temperature of 50 °C on their interactions with free radicals. The kinetics of the interactions of the extracts with DPPH were assessed using electron paramagnetic resonance (EPR) spectroscopy. The sea buckthorn and European cranberry extracts quench the EPR signal of DPPH free radicals, which indicates their antioxidant potential. The EPR method further showed that a mixture of sea buckthorn and cranberry extracts in a volume ratio of 2:1 was more potent in quenching free radicals compared to a mixture of these extracts in a ratio of 1:2. Our findings demonstrate that long-term UVA radiation exposure reduces the ability of sea buckthorn and cranberry extracts to interact with free radicals. Moreover, storage at elevated temperatures does not affect the interaction of sea buckthorn extract with free radicals, while it alters the ability of cranberry extract to interact with free radicals. This study has demonstrated that an important factor in maintaining the ability to scavenge radicals is the storage of raw materials under appropriate conditions. *H. rhamnoides* and *V. oxycoccos* extracts can be used as valuable raw materials with antioxidant properties in the pharmaceutical and cosmetic industries.

## 1. Introduction

Plant extracts, due to the richness of natural antioxidants, are not only skin care ingredients but also components that affect the stability, durability, and usefulness of products applied topically to the skin [1,2]. It is known that storage conditions, such as light and temperature, have a significant impact on the antioxidant properties of plant raw materials [3,4]. One of the requirements for antioxidants used in topically applied preparations on the skin is their resistance to physical factors such as light or temperature. This is important because under the influence of UV light or increased temperature, the functional qualities of cosmetic raw materials decrease. Free radicals and free radical reaction products formed under the influence of UV radiation or elevated temperatures can initiate adverse product changes and may also contribute to skin irritation or local inflammation [4,5,6,7]. For this reason, the pharmaceutical and cosmetic industries are looking for new active ingredients with antioxidant properties.

The fruit of the sea buckthorn (*Fructus Hippophaës*) and the fruit of the cranberry (*Fructus Oxycocci*) are valuable herbal raw materials used in the pharmaceutical and cosmetic industries [8,9]. *Hippophaë rhamnoides* L., known as sea buckthorn, is a highly branched shrub or a small tree belonging to the Elaeagnaceae family [8]. European cranberry (*Vaccinium oxycoccos* L., syn. *Oxycoccus palustris* Pers.) is a low, creeping shrub belonging to the Ericaceae family [10]. The use of sea buckthorn in medicine, pharmacy, and cosmetics is well known and documented. Preparations based on sea buckthorn are available in various forms, e.g., liquids, powders, tablets, suppositories, pastes, ointments, aerosols, or patches [8,11]. Sea buckthorn is a plant that for centuries has been used as a medicinal raw material, a nutritional product, and a natural skin care agent. Sea buckthorn in the form of infusions has been used externally in skin diseases, for burns, for dry skin, and as a softening cosmetic agent; its oil added to cream has been applied as a radiation protective agent [12]. Currently, sea buckthorn is gaining importance as an active ingredient of pharmaceuticals and cosmeceuticals [12,13,14]. The beneficial effects of sea buckthorn fruit extract on skin have been reported [14,15]. Due to its high content of antioxidants and nutrients, sea buckthorn is used in phytopharmaceuticals as well as in moisturising, revitalising, restorative, and radiation protective cosmetics [11,13]. Moreover, recent studies have confirmed and expanded the possibilities of traditional sea buckthorn use for the treatment of dermatological diseases (including psoriasis, ichthyosis, lupus erythematosus, rosacea or common acne, and atopic dermatitis) [8,13,16], which is associated with the anti-inflammatory, antimicrobial, radioprotective, and wound healing effects of this raw material [8,11,16,17]. Attention is also paid to the antioxidant properties of sea buckthorn and its ability to scavenge free radicals [16,18,19]. Research on the biological properties of cranberry fruit also indicates growing interest in the use of this raw material in the pharmaceutical and cosmetic industries. Based on the available literature, it can be concluded that the European cranberry has antibacterial, antiviral, antifungal, anti-inflammatory, and anticancer properties, displaying antioxidant and radical scavenging activity [19,20]. It has been shown that cranberry extract has strong DPPH radical scavenging activity and the ability to inhibit hydroxyl radicals [20]. *V. oxycoccos* extract can potentially be used in topical preparations that protect against free radicals, which adversely affect not only the condition of the skin but also the quality of pharmaceutical and cosmetic products [19].

Several analytical methods have been used to assess the antioxidant activity of sea buckthorn [18,19,21] and European cranberry [19,22]. To the best of our knowledge, this is the first time that the free radical interaction ability of glycolic extracts from *Hippophaë rhamnoides* and *Vaccinium oxycoccos* fruits was measured by electron paramagnetic resonance (EPR) spectroscopy. The present study aimed to evaluate the DPPH radical scavenging activity of *H. rhamnoides* and *V. oxycoccos* extracts, as well as the effect of UVA radiation and a temperature of 50 °C on these properties.

## 2. Results

### 2.1. Free Radical Interactions of Hippophaë rhamnoides and Vaccinium oxycoccos Fruit Glycolic Extracts Stored at Room Temperature

**Figure 1:**
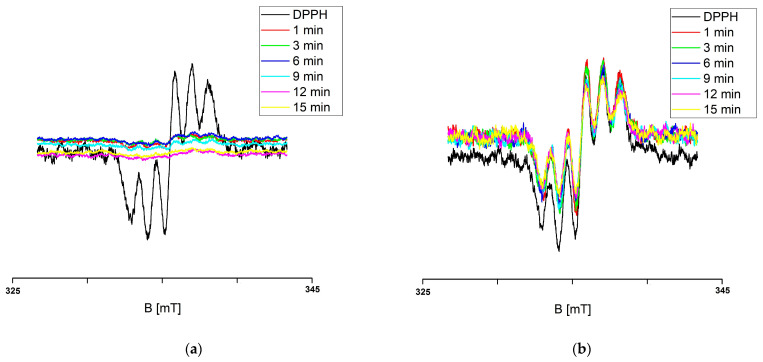
EPR spectra of DPPH free radicals interacting with (**a**) *H. rhamnoides* and (**b**) *V. oxycoccos* glycolic extracts stored at room temperature for interaction times of 1, 3, 6, 9, 12, and 15 min. B: magnetic induction.

The studies carried out in the present paper demonstrated the ability of *H. rhamnoides* and *V. oxycoccos* extracts to quench free radicals. Figure 1 shows the EPR spectra of DPPH free radicals in contact with sea buckthorn and European cranberry extracts recorded for different interaction times of 1, 3, 6, 9, 12, and 15 min.

**Figure 2:**
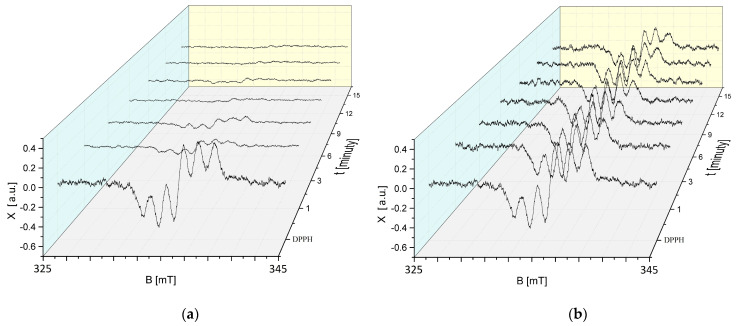
Effect of interaction time (t) of DPPH free radicals with (**a**) *H. rhamnoides* and (**b**) *V. oxycoccos* glycolic extracts stored at room temperature on EPR spectra of DPPH. B: magnetic induction; X: EPR signal height of DPPH at the point of magnetic induction B.

The tested extracts are characterised by antioxidant properties, as shown by the recorded decrease in the EPR spectra. The EPR spectra of DPPH free radicals upon interaction with sea buckthorn and cranberry extracts stored at room temperature decrease with the increasing contact time of DPPH with the extracts, which corresponds to the quenching of an increasing number of free radicals. The EPR spectra of DPPH free radicals after reaching a minimum amplitude value do not change in magnitude, corresponding to a stabilisation of the interactions (Figure 2).

**Figure 3:**
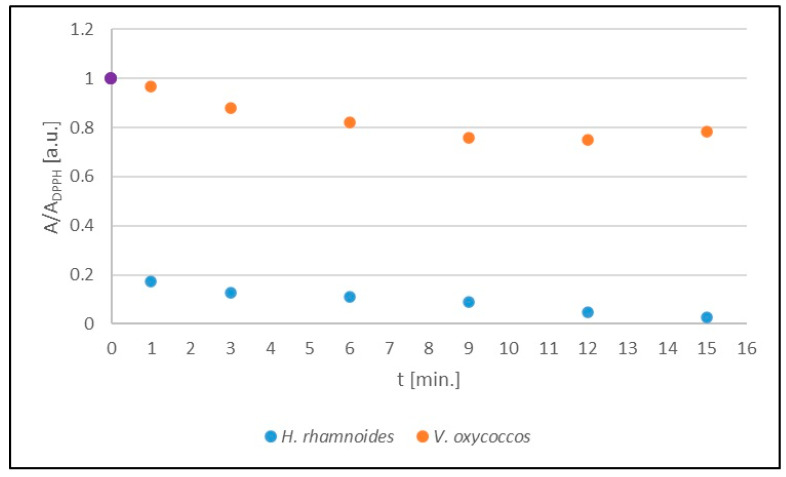
Kinetics of the interaction of DPPH free radicals with *H. rhamnoides* and *V. oxycoccos* glycolic extracts stored at room temperature shown as the change in amplitude (A/A_DPPH_) of the EPR lines of DPPH free radicals with increasing time (t) of interaction with the extract. A: EPR line amplitude of DPPH free radicals in contact with the extract under study; A_DPPH_: EPR line amplitude of DPPH in standard solution. For the standard solution, the amplitude (A/A_DPPH_) is 1 (purple dot).

The kinetics of the interaction of DPPH free radicals with sea buckthorn and European cranberry extracts stored at room temperature are illustrated in Figure 3. The amplitudes (A/A_DPPH_) of the EPR spectra of DPPH, expressed as the ratio of the amplitude value (A) of the EPR spectrum of free radicals in contact with the tested extract to the amplitude value (A_DPPH_) of the standard EPR spectrum of DPPH for the glycol extracts tested, take values less than 1, which is characteristic of antioxidants. The antioxidant quenches the DPPH free radicals, resulting in reduced microwave absorption and a lowering of the amplitude (A) value of the EPR line relative to the amplitude (A_DPPH_) of the DPPH line in the standard solution. Stronger interactions of the DPPH free radicals with the antioxidant are accompanied by a greater decrease in the amplitude value (A) of the DPPH EPR line and thus a greater decrease in the amplitude value (A/A_DPPH_). The magnitudes of the interactions of the tested extracts with free radicals were compared by analysing the values of the amplitudes (A/A_DPPH_) of the DPPH EPR lines in the curves showing the kinetics of the interactions. The amplitudes (A/A_DPPH_) of the EPR DPPH lines with increasing interaction time (t) with the tested extracts initially decrease with increasing time, reach a minimum value, and then no longer change with increasing time.

### 2.2. Free Radical Interactions of a Mixed Hippophaë rhamnoides and Vaccinium oxycoccos Fruit Glycolic Extract at Different Volume Ratios Stored at Room Temperature

**Figure 4:**
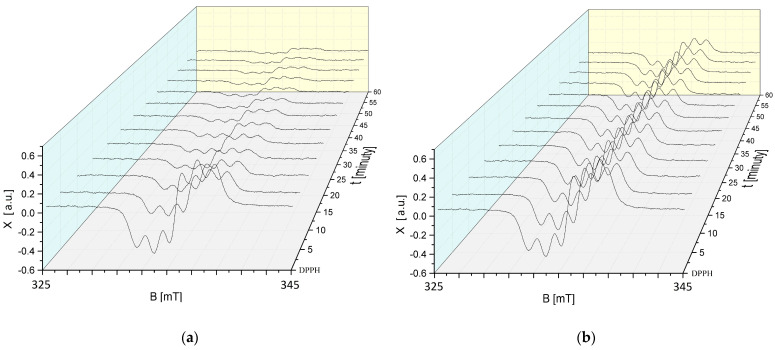
Effect of interaction time (t) of DPPH free radicals with a mixture of *H. rhamnoides* and *V. oxycoccos* extracts in volume ratios of (**a**) 2:1 and (**b**) 1:2 stored at room temperature on the EPR spectra of DPPH. B: magnetic induction; X: height of the DPPH EPR signal at the point of magnetic induction B.

**Figure 5:**
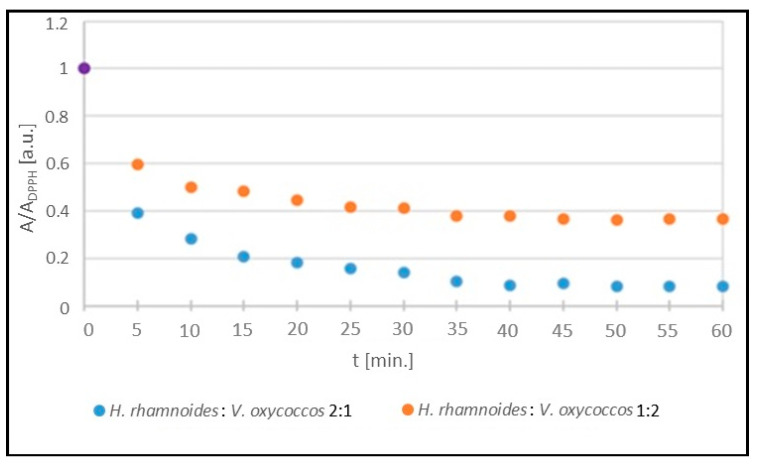
Kinetics of the interaction of DPPH free radicals with mixtures of *H. rhamnoides* and *V. oxycoccos* extracts at volume ratios of 2:1 and 1:2 shown as the change in the amplitude (A/A_DPPH_) of the EPR lines of DPPH free radicals with increasing time (t) of interaction. A: amplitude of the EPR line of DPPH free radicals in contact with the test extract mixture; A_DPPH_: amplitude of the EPR line of DPPH in the standard solution. For the standard solution, the relative amplitude (A/A_DPPH_) is 1 (purple dot).

**Figure 6:**
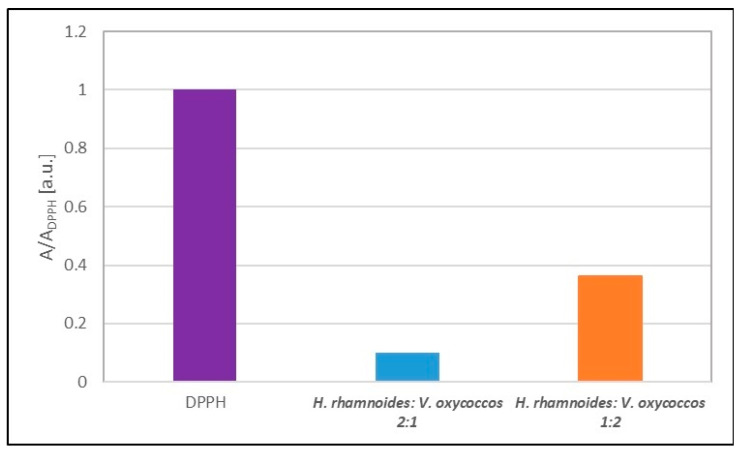
Comparison of the amplitudes (A/A_DPPH_) of the EPR spectra of DPPH free radicals interacting for 60 min with mixtures of *H. rhamnoides* and *V. oxycoccos* extracts at volume ratios of 2:1 and 1:2. A: amplitude of the EPR line of DPPH free radicals in contact with the test extract mixture; A_DPPH_: amplitude of the EPR line of DPPH in the standard solution. For the standard solution, the relative amplitude (A/A_DPPH_) is 1 (purple pistil).

Studies using EPR spectroscopy of mixtures containing both sea buckthorn and cranberry extracts in volume ratios of 2:1 and 1:2, stored at room temperature, confirmed the interactions of the extract mixtures with free radicals and the dependence of the magnitude of these interactions on the content of the individual components. The effect of the interaction time (t) of DPPH free radicals with a mixture of sea buckthorn and cranberry extracts in volume ratios of 2:1 and 1:2 on the EPR spectra of DPPH is shown in Figure 4a and Figure 4b, respectively. The kinetics and magnitude of the interactions of mixtures of extracts with DPPH are compared in Figure 5 and Figure 6.

EPR studies confirmed the quenching of DPPH free radicals by mixtures of extracts in volume ratios of 2:1 and 1:2 stored at room temperature. These mixtures quenched the DPPH free radicals and their EPR spectra (Figure 4a,b). The interaction kinetics of the two extract mixtures are similar (Figure 5); however, the minimum values of the amplitudes (A/A_DPPH_) of the EPR spectra of the DPPH free radicals for these mixtures differ (Figure 6). A smaller value of the amplitude (A/A_DPPH_) of the EPR lines of the DPPH free radicals was obtained for a mixture of sea buckthorn and cranberry extracts in a volume ratio of 2:1, compared to a mixture in a volume ratio of 1:2 (Figure 6). The mixture of extracts in a volume ratio of 2:1 quenches DPPH free radicals more strongly and is therefore a more potent antioxidant.

### 2.3. Free Radical Interactions of Hippophaë rhamnoides and Vaccinium oxycoccos Fruit Glycolic Extracts Exposed to UVA Radiation 

**Figure 7:**
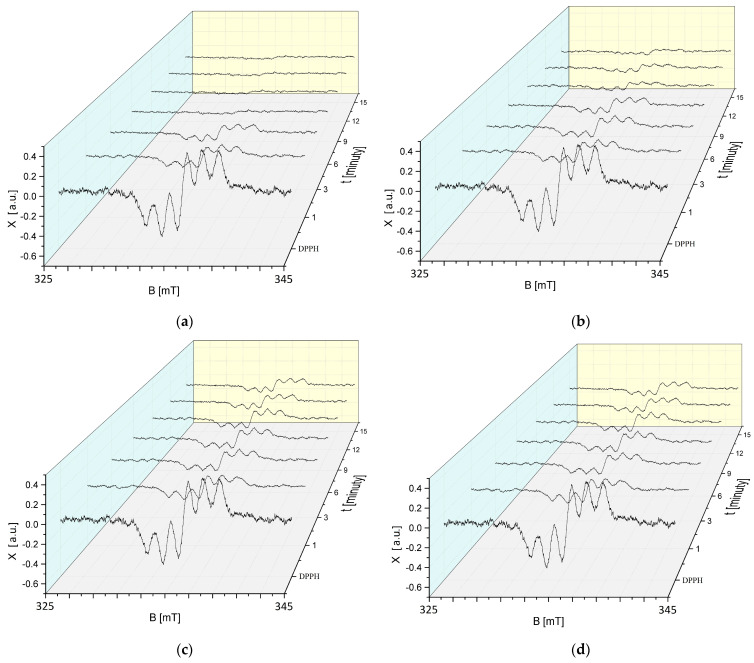
Effect of interaction time (t) of DPPH free radicals with *H. rhamnoides* extract on EPR spectra of DPPH. Results for extract treated with UVA for (**a**) 30 min; (**b**) 60 min; (**c**) 90 min; and (**d**) 120 min. B: magnetic induction; X: height of EPR DPPH signal at the point of magnetic induction B.

The effect of the interaction time (t) of DPPH free radicals with sea buckthorn extract on the EPR spectra of DPPH in contact with the extract exposed to UVA for 30, 60, 90, and 120 min is shown in Figure 7a–d. The EPR spectra of DPPH free radicals with a g-factor of 2.0036 for all UVA exposure times of *H. rhamnoides* extract decrease due to the interaction with the extract, indicating that the antioxidant properties are retained after UVA exposure. After an initial decrease in the EPR spectra of DPPH free radicals interacting with UVA-treated sea buckthorn extract, the spectra stabilise. Changes in the EPR spectra of DPPH free radicals depend on the exposure time of sea buckthorn extract to UVA radiation.

**Figure 8:**
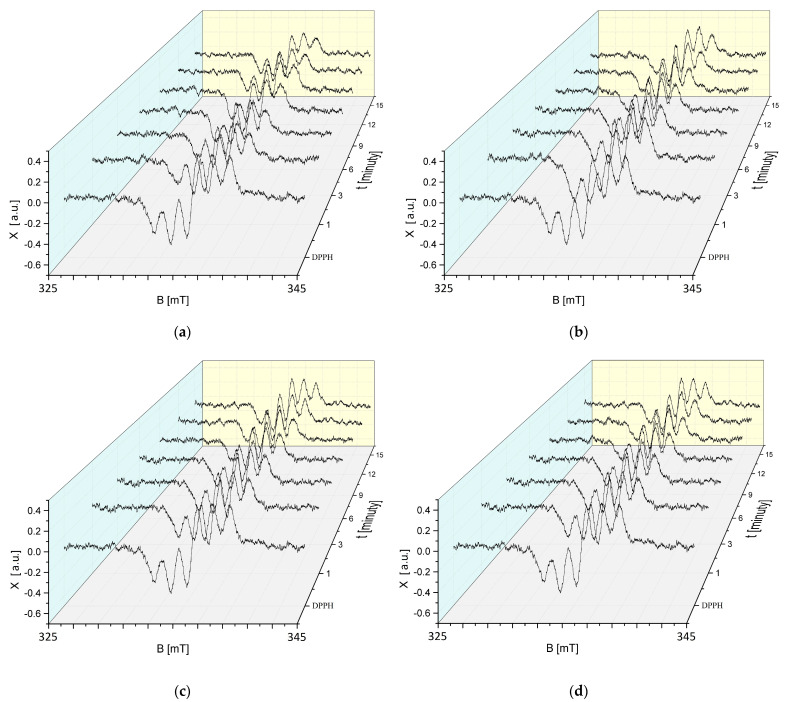
Effect of interaction time (t) of DPPH free radicals with *V. oxycoccos* extract on EPR spectra of DPPH. Results for extract treated with UVA for (**a**) 30 min; (**b**) 60 min; (**c**) 90 min; and (**d**) 120 min. B: magnetic induction; X: height of the EPR signal of DPPH at the point of magnetic induction B.

Studies using EPR spectroscopy have shown that the EPR spectra of DPPH free radicals decrease when exposed to UVA-treated European cranberry extract (Figure 8a–d), confirming the preservation of its antioxidant properties after exposure to this radiation.

**Figure 9:**
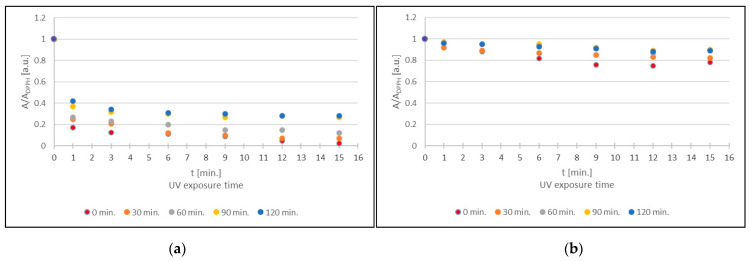
Kinetics of the interaction of DPPH free radicals with glycolic extracts from (**a**) *H. rhamnoides* and (**b**) *V. oxycoccos* shown as the change in amplitude (A/A_DPPH_) of DPPH EPR spectra with increasing time (t) of interaction with the extract. Results for extracts exposed to UVA for 30 min; 60 min; 90 min; and 120 min. For the standard solution, the amplitude (A/A_DPPH_) is 1 (purple dot). A: EPR line amplitude of DPPH free radicals in contact with the test extract; A_DPPH_: EPR line amplitude of DPPH in the standard solution.

Figure 9 shows the kinetics of the interaction of DPPH free radicals with sea buckthorn and European cranberry glycolic extracts after the exposure of the extract to UVA radiation for (a) 30 min, (b) 60 min, (c) 90 min, and (d) 120 min. The nature of the changes in the amplitude (A/A_DPPH_) of the EPR lines of the DPPH free radicals with the increasing time (t) of interaction with the extract is similar for sea buckthorn (Figure 9a) and cranberry (Figure 9b) extracts not exposed to UVA radiation (0 min) and for extracts exposed to UVA radiation for 30, 60, 90, and 120 min. The rates of free radical interactions of these extracts are similar. Initially, a decrease in the amplitude value (A/A_DPPH_) of the EPR line of DPPH free radicals is observed with the increasing time (t) of interaction with the extract, and then, after reaching a minimum value, the amplitude value (A/A_DPPH_) of the EPR line is constant.

**Figure 10:**
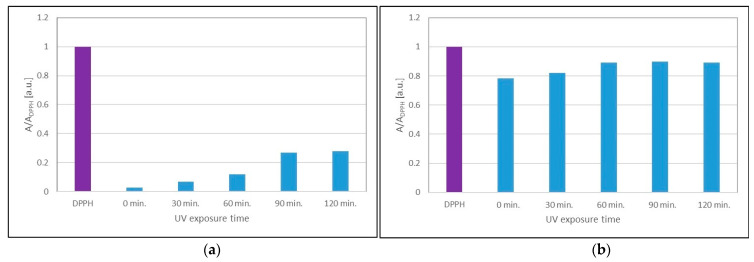
Comparison of the amplitudes (A/A_DPPH_) of the EPR spectra of DPPH free radicals interacting for 15 min with glycolic extract of (**a**) *H. rhamnoides* and (**b**) *V. oxycoccos* not treated with UVA (0 min) and with UVA-treated extracts for 30, 60, 90, and 120 min. For the standard solution, the amplitude (A/A_DPPH_) is 1 (purple pistil). A: amplitude of the EPR line of the DPPH free radicals in contact with the test extract; A_DPPH_: amplitude of the EPR line of DPPH in the standard solution.

It was predicted that UVA radiation could negatively affect the antioxidant properties of the extracts. The EPR method showed that UVA radiation decreases the antioxidant capacity of sea buckthorn and cranberry extracts (Figure 10) after prolonged exposure of the extracts to this radiation. The effect of UVA radiation on the lower quenching of DPPH free radicals by the tested extracts results in an increase in the minimum amplitude values (A/A_DPPH_) of the EPR spectra of DPPH. The minimum amplitude values (A/A_DPPH_) of the EPR lines of DPPH free radicals interacting with sea buckthorn (Figure 10a) and cranberry (Figure 10b) extracts after UVA exposure for 60, 90, and 120 min are greater than the minimum value (A/A_DPPH_) for the untreated UVA extracts. This effect was not observed for the shorter duration of UVA radiation (30 min) on cranberry extract (Figure 7b).

### 2.4. Free Radical Interactions of Hippophaë rhamnoides and Vaccinium oxycoccos Fruit Glycolic Extracts Exposed to a Temperature of 50 °C 

**Figure 11:**
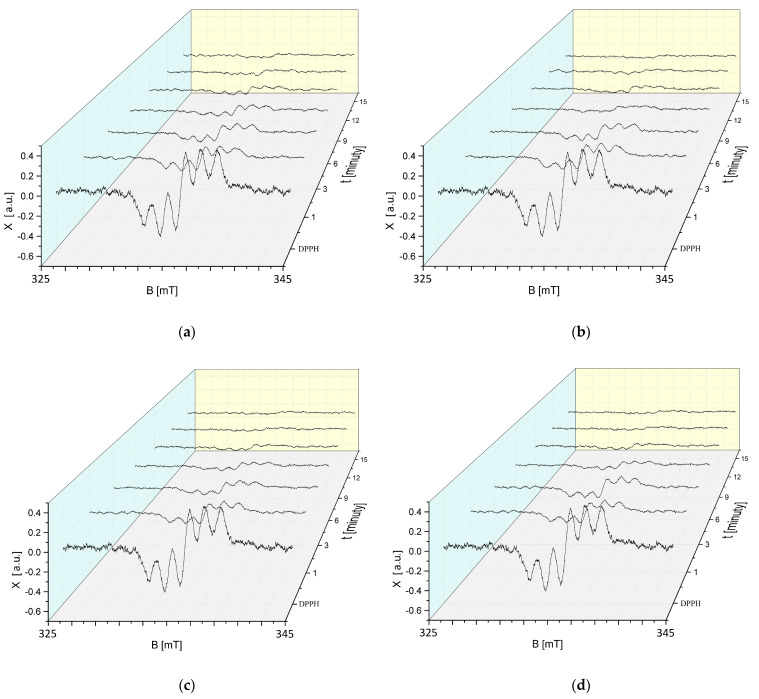
Effect of interaction time (t) of DPPH free radicals with sea buckthorn extract on EPR spectra of DPPH. Results for *H. rhamnoides* extract treated at 50 °C for (**a**) 30 min; (**b**) 60 min; (**c**) 90 min; and (**d**) 120 min. B: magnetic induction; X: height of DPPH EPR signal at the point of magnetic induction B.

The changes in the EPR spectra of DPPH free radicals with increasing interaction time (t) with sea buckthorn extract stored at 50 °C for 30, 60, 90, and 120 min are shown in Figure 11a–d. The EPR spectra of DPPH free radicals decrease with the increasing interaction time with sea buckthorn extract stored at an elevated temperature, and then, their magnitude does not change with increasing time. Such relationships were found for all storage times of sea buckthorn extract at 50 °C (Figure 11a–d).

**Figure 12:**
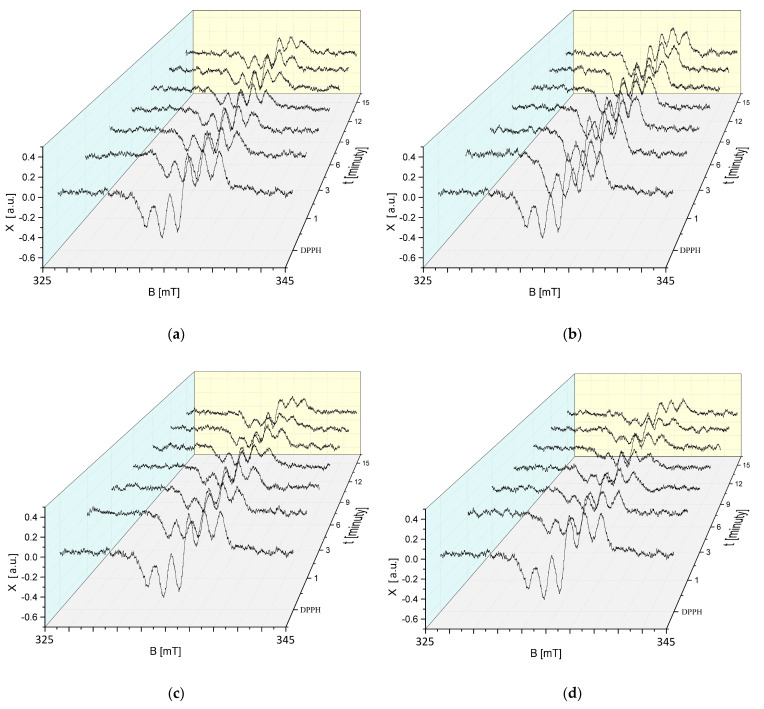
Effect of interaction time (t) of DPPH free radicals with *V. oxycoccos* extract on EPR spectra of DPPH. Results for extract treated at 50 °C for (**a**) 30 min; (**b**) 60 min; (**c**) 90 min; and (**d**) 120 min. B: magnetic induction; X: height of DPPH EPR signal at the point of magnetic induction B.

Studies using EPR spectroscopy showed that regardless of the time for which the cranberry extract was stored at an elevated temperature of 50 °C, it still showed antioxidant properties. In Figure 12a–d, a reduction in the magnitude of the EPR spectra can be observed, indicating the quenching of the DPPH free radicals by the test extract.

**Figure 13:**
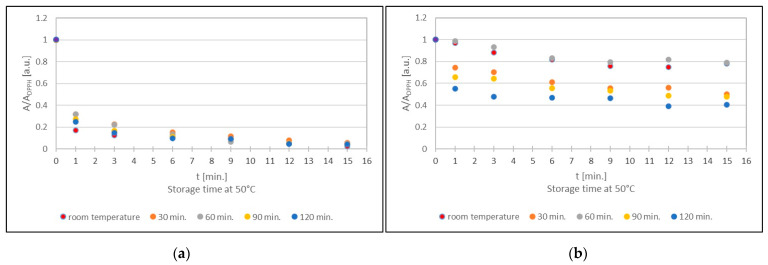
Effect of elevated storage temperature on the kinetics of interaction of DPPH free radicals with extract from (**a**) *H. rhamnoides* and (**b**) *V. oxycoccos*, shown as the change in amplitude (A/A_DPPH_) of the EPR lines of DPPH free radicals with increasing time (t) of interaction with the extract. A: EPR line amplitude of DPPH free radicals in contact with the test extract; A_DPPH_: EPR line amplitude of DPPH in the standard solution. For the standard solution, the amplitude (A/A_DPPH_) is 1 (purple dot). Results for extracts stored at room temperature and for extracts stored at 50 °C for 30, 60, 90, and 120 min.

The kinetics of the interaction of DPPH free radicals from sea buckthorn and cranberry as the change in the amplitude (A/A_DPPH_) of the EPR lines of DPPH free radicals with the increasing time (t) of interaction with the extracts stored at room temperature and at 50 °C for 30 min, 60 min, 90 min, and 120 min are shown in Figure 13. The amplitude (A/A_DPPH_) of the EPR lines of the DPPH free radicals decreases with the increasing interaction time for sea buckthorn extracts, irrespective of the storage time of the extract at 50 °C. The points showing the kinetics on the graphs do not differ significantly. The increased storage temperature (50 °C) of the extract does not change the kinetics of its interaction with DPPH free radicals (Figure 13a). The values of the amplitude (A/A_DPPH_) of the EPR lines for storage times of 30 min, 90 min, and 120 min for the cranberry extract at 50 °C are smaller than those for the extract stored at room temperature. This effect is not observed for cranberry extract stored at 50 °C for 60 min (Figure 13b).

**Figure 14:**
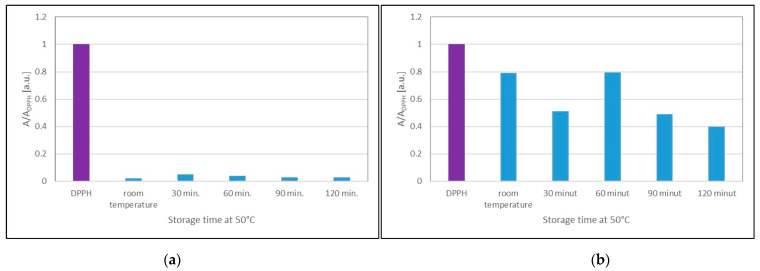
Comparison of the amplitudes (A/A_DPPH_) of the EPR spectra of DPPH free radicals interacting for 15 min with (**a**) *H. rhamnoides* and (**b**) *V. oxycoccos* extract stored at room temperature at 50 °C for 30, 60, 90, and 120 min. A: EPR line amplitude of DPPH free radicals in contact with the extract under test; A_DPPH_: EPR line amplitude of DPPH in the standard solution. For the standard solution, the relative amplitude (A/A_DPPH_) is 1 (purple pistil).

There was no effect of storing sea buckthorn extract at an elevated temperature of 50 °C for 30 min, 60 min, 90 min, and 120 min on the amplitude value (A/A_DPPH_) of the DPPH free radical EPR lines (Figure 14a). Increasing the storage temperature of sea buckthorn extract from room temperature to 50 °C did not change its antioxidant effects. The minimum values of the amplitudes (A/A_DPPH_) of the EPR spectra of DPPH free radicals interacting with cranberry extracts stored at room temperature and at 50 °C shown in Figure 14b indicate that storing cranberry extract at 50 °C decreases the A/A_DPPH_ values for storage times of 30 min, 90 min, and 120 min. This effect indicates an increase in the antioxidant properties of the extract for these storage conditions.

## 3. Discussion

Intensive research on free radical processes, which has been carried out for many years, contributes to a better understanding of these phenomena and allows us to notice their complexity. A high level of radicals in the absence of effective antioxidant mechanisms can cause damage to cellular structures and the oxidation of lipids, proteins, and nucleic acids. Furthermore, while exhibiting the natural defence mechanisms against the action of radicals, in cases of excessive free radical production, the skin is exposed to the effects of oxidative stress [23]. Free radicals, by disrupting the defence mechanisms, regulation, and reconstruction mechanisms, contribute significantly to skin damage and faster aging. 

Free radicals adversely affect not only the condition of the skin but also the quality of products applied topically to the skin. Free radicals and free radical reaction products formed under the influence of UV radiation or elevated temperature may initiate unfavourable changes, e.g., consistency, colour, and smell of the products. In particular, products containing fatty substances, due to the presence of unsaturated bonds in their structure, easily undergo autooxidation processes. Autooxidation products can contribute to skin irritation, allergies, or local inflammation and have mutagenic and carcinogenic effects. Oxidation reactions can occur during both the preparation and storage of already finished formulations. To reduce the negative impact of external factors on the quality of products for external use/topically applied to the skin, various protective procedures are used, e.g., limiting the access of oxygen and light, storing products at low temperatures, using airtight packages, and eliminating oxidising or oxidation-accelerating compounds from the environment [5,24]. Another way to prevent the autooxidation of preparations is to use antioxidants, both synthetic and natural [5,23]. Hence, the pharmaceutical and cosmetic industries are looking for new active ingredients with antioxidant properties. 

The research carried out in this paper with the use of EPR spectroscopy extended the existing knowledge on the interactions of plant extracts with free radicals. The present study conducted using EPR spectroscopy and model DPPH free radicals showed the capacity of *Hippophae rhamnoides* and *Vaccinium oxycoccos* fruit glycolic extracts to quench free radicals. As a result of interactions with the sea buckthorn and European cranberry extracts stored at room temperature, the EPR spectra of DPPH free radicals decrease as the time of DPPH–extract contact increases, which corresponds to the quenching of an increasingly high number of free radicals. The results of our study are confirmed by the findings of studies using spectrophotometric methods of measuring the antioxidant activity of plant extracts. The antioxidant activity of *H. rhamnoides* extracts has been evaluated using tests such as the ferric reducing antioxidant power (FRAP) assay [17,19], ferric thiocyanate method [22], and metal chelating activities [22] and also defined as the ability to scavenge various free radicals, i.e., ABTS^•+^ radical cation [19,25], stable DPPH radical [17,21,25], superoxide anion radical (O_2_^•−^) [17,18,21,25], hydroxyl radical (^•^OH) [17,18,21,25], and nitric oxide (NO^•^) radical [17,18,25]. The antioxidant potential of *V. oxycoccos* fruit glycolic extract was tested using 2,2′-azino-bis(3-ethylbenzothiazoline-6-sulfonic acid) (ABTS^•+^) radical-cation-based assays, as well as the ferric reducing antioxidant power (FRAP) assay, and was expressed as the Trolox equivalent (TEAC). The TEAC values for cranberry fruit extract were 0.73 μmol Trolox/mL extract (ABTS) and 0.39 μmol Trolox/mL extract (FRAP) [19]. Other authors, using spectrophotometric methods, assessed the antioxidant activity of cranberry fruit as the efficiency in quenching the stable DPPH radical and the hydroxyl radical scavenger ability [20].

The literature data have revealed that the antioxidant effect of plant extracts is largely associated with the content of polyphenols capable of scavenging free radicals, including the DPPH radical, peroxide anion radical (O_2_^•−^), nitric oxide (NO^•^). and hydroxyl radical (^•^OH), as well as the chelation of transition metal ions [17,18,21,22,25].

The available data indicate that the scavenging activity of sea buckthorn is related to the content of antioxidants such as flavan-3-ols ((+)-catechin, (−)-epicatechin, (−)-epi-catechin-gallate, (−)-epigallo-catechin), proanthocyanidins, flavonols (myricetin, quercetin, kaempferol, isorhamnetin), and phenolic acids (including hydroxybenzoic acid derivatives (salicylic, protocatechuic, gallic, 2,5-dihydroxybenzoic, pyrocatechuic, vanillic acid) and hydroxycinnamic acid derivatives (caffeic, m-coumaric, o-coumaric, p-coumaric, caffeic acid) [17,18,22,26,27]. In cranberry fruit, on the other hand, the free radical scavenging capacity is attributed to compounds such as phenolic acids (benzoic, *p*-coumaric, chlorogenic, caffeic, ferulic, gentisic acid) and flavonols (quercetin, myricetin, kaempferol), as well as anthocyanins (peonidin-3-galactoside, cyanidin-3-arabinoside, cyanidin-3-galactoside, peonidin-3-arabinoside, peonidin-3-glucoside, and cyanidin-3-glucoside) [10,19,20,28]. These compounds, due to the presence of a hydroxyl group on an aromatic ring, act as reducing agents and antioxidants because they interrupt chain oxidation reactions by donating a hydrogen atom or chelating metals [10,17,18,21,25]. Data on the evaluation of selected groups of polyphenolic compounds of sea buckthorn and *European cranberry* fruit glycolic extracts tested in this study, included in our previous publication [19], indicate that the total polyphenol content in 100 mL (calculated as gallic acid equivalent) of the sea buckthorn fruit extract was 60.85 mg, while that of cranberry extract was 21.39 mg. Flavonols have been demonstrated to dominate among different polyphenolic compounds, constituting about 28% of the polyphenols present in the sea buckthorn extract, while proanthocyanidins are predominant in the cranberry extract [19]. Another study has shown a total phenolic content in an aqua-methanolic extract of sea buckthorn of 84.28 mg of gallic acid equivalent (GAE)/g of extract [18]. A study on *H. rhamnoides* alcoholic extracts revealed a total content of phenolics of 28.84 mg/100 mL, calculated using the tannic acid equivalent [21]. Furthermore, the total phenolic content of 100% methanolic sea buckthorn extract has been found to be 302.72 mg of GAE/gm of extract [17]. The total polyphenol content of the glycolic extract from sea buckthorn fruit has been shown to be almost three times higher than that of the cranberry fruit extract [19], as reflected in the greater decrease in amplitude (A/A_DPPH_) values recorded in this study using EPR spectroscopy. The obtained results are in line with those of Velioglu et al. [29], which indicate that the antioxidant activity of fruit is mainly related to the polyphenol content. This is also reflected in studies on sea buckthorn and cranberry fruit, in which a high content of compounds from the polyphenol group corresponds to antioxidant properties [10,28].

The literature review shows that antioxidant properties depend not only on the content of polyphenolic compounds but also on the type of extract and the extraction time. Muzykewicz et al. [30] evaluated the effect of the extractant (ethanol, methanol, acetone) and the extraction time on the antioxidant properties of *H. rhamnoides* extracts obtained by ultrasonic extraction. One-hour extraction in methanol has been shown to be the most effective method for obtaining sea buckthorn extracts with good antioxidant capacity [30]. According to the study by Kant et al. [17], 100% methanolic sea buckthorn extract exhibited the highest free radical scavenging ability among the extracts tested (100% methanolic, 70% methanolic, 100% aqueous). The study by Chauhan et al. [25], evaluating sea buckthorn seed oil and different extracts (100% methanolic, 70% ethanolic, 50% methanolic and 100% aqueous), revealed that 70% ethanolic sea buckthorn extract shows the best in vivo and in vitro antioxidant activity. According to Chaman et al. [31], the methanol extract showed the maximum antioxidant potential, followed by extracts of water, petroleum ether, and chloroform. The findings reported by Papuc et al. [21] indicate that the alcoholic extract of sea buckthorn exhibits a free radical scavenging capacity and significantly reduces the malondialdehyde content, which is a measure of lipid peroxidation and shows the antioxidant activity. Moreover, the results reported by Büyükokuroğlu and Gulcin [22] clearly demonstrated the antioxidant and antiradical activities of hexanoic sea buckthorn fruit extracts. In another study concerning the antioxidant activity of water–glycerine sea buckthorn extracts prepared from fresh and dried fruits, the authors demonstrated that fresh fruit extracts exhibit the highest free radical scavenging capacity, which depends on the amount of fruit used, the type of fruit, their degree of botanical ripeness, and the processing method [32].

The antioxidant properties of plant extracts are influenced by the presence of the various active compounds they contain [29]. Individual components may interact to exhibit one of the possible effects: synergistic (when the combined effect of two or more components is greater than the sum of the effects of each component used alone), additive (when the combined effect of two or more components is equal to the sum of the effects of each component used alone), or antagonistic (when the combined effect of two or more components is less than the sum of the effects of each component used alone) [33,34]. Previous findings on antioxidant interactions show the possibility of polyphenolic compounds interacting with other compounds with antioxidant properties, e.g., the synergism of myricetin and tocopherol [33]. Romano et al. [35] demonstrated the synergism of rosemary extract and ascorbic acid, tocopherol, and BHA in a DPPH radical assay. Some data on the synergistic antioxidant activity of plant extracts are also available in the literature [34,36]. Jain et al. [36] showed that green tea extract showed better antioxidant properties when combined with other extracts, which are important sources of polyphenolic compounds, including flavonoids. Based on the study, the authors concluded that a mixture of plant extracts, including *Vitis vinifera* ethanol–water extract, *Phyllanthus emblica* methanolic extract, *Punica granatum* aqueous extract, *Cinnamomum cassia* ethanol–water extract, and *Ginkgo biloba* methanolic extract, acted synergistically with *Camellia sinensis* aqueous extract [36]. Vattem et al. [37] observed synergism for mixtures of cranberry extract with blueberry, oregano, and grape seed extracts with regard to antioxidant properties. For cosmetic products based on natural plant extracts, both the interactions between individual antioxidant active compounds and between the plant extracts used in the formulation are important. A composition of two or more antioxidants may act more effectively than single antioxidants added to cosmetic products (synergism) or less effectively if the ingredients with antioxidant properties act antagonistically to each other. Due to the fact that not only components of plant extracts but also crude extracts are added to cosmetic products, it seems important to study the interactions between plant extracts. In addition, this research is of considerable practical importance, as knowledge in this area can be useful in the development of formulations containing raw materials of plant origin and the design of products with more beneficial properties. This study was undertaken to determine the effect of the volume content of sea buckthorn and European cranberry extracts in a mixture of these extracts on their interactions with free radicals. A decrease in the EPR spectra due to the interaction with the mixture of extracts was observed for sea buckthorn and cranberry extracts in volume ratios of 2:1 and 1:2, confirming their antioxidant character. The kinetics of the free radical interactions of these two extracts (2:1 and 1:2) are similar. The tested extract mixtures differ in the magnitude of free radical interactions. The mixture of sea buckthorn and cranberry extracts in a volume ratio of 2:1 is a more potent antioxidant than the mixture of sea buckthorn and cranberry extracts in a volume ratio of 1:2 because it causes greater quenching of the EPR spectra, for which smaller values of the minimum amplitude (A/A_DPPH_) were obtained. Considering the spectroscopic parameters, it was found that mixtures of glycol extracts of sea buckthorn and bog cranberry in volume ratios of 2:1 and 1:2 quenched the EPR DPPH spectra more strongly than European cranberry extract but less strongly than sea buckthorn extract. Further studies are needed to verify synergism in the antioxidant activity of plant extracts. The results obtained in this study showed that the antioxidant potential of a mixture of plant extracts can be increased by selecting appropriate volume ratios of the component extracts in the mixture. Glycolic extracts of sea buckthorn and European cranberry and mixtures of these extracts can be used in cosmetic preparations, with a higher content of sea buckthorn extract increasing the antioxidant effects of the product.

Currently, new active ingredients are being sought that are not only beneficial for skin care but also help to neutralise the negative effects of free radicals on the skin and on preparations applied topically [5]. The use of antioxidants in cosmeceuticals and pharmaceuticals is necessary and fully justified, as it prolongs the shelf life and affects the quality of products, e.g., of the emulsions which are extensively used as a vehicle in the delivery of drugs and active substances across the skin [24]. The antioxidants used most frequently are tocopherols, ascorbyl palmitate, butylated hydroxyanisole (BHA), and butylated hydroxytoluene (BHT) [5,38]. Recently, plant extracts used as broad-spectrum ingredients have also gained much attention. The use of well-composed mixtures of raw materials of plant origin may improve the effectiveness of other antioxidants and reduce the harmful effects of synthetic antioxidants (such as BHT) on human skin [34,38,39]. The phytoconstituents contained in plant extracts play an important role in free radical scavenging; however, as the research results show, storage conditions such as light and temperature have a significant impact on the antioxidant properties of plant raw materials [3,4]. One of the requirements for antioxidants used in preparations applied topically to the skin is their resistance to physical factors, such as light or temperature. It is worth noting that free radicals and products of free radical reactions formed under the influence of UV radiation and increased temperature or in the presence of traces of heavy metal ions (copper, iron, cobalt, manganese, chromium) reduce the quality and utility values of cosmetic and pharmaceutical raw materials [4,5,7]. Under the conditions of intense sunlight, the molecules undergo diverse photochemical reactions. Complex free radical reactions with the participation of the generated free radicals may accompany the UV irradiation of the test sample. As a result of the absorption of UV radiation energy, the structure of the molecule can be changed and transformed into a paramagnetic particle with unpaired electrons [40]. There are known research results indicating the negative impact of ultraviolet radiation on the antioxidant properties of bioactive compounds such as polyphenols, ascorbic acid, anthocyanins [41], and carotenoids, including β-carotene, lycopene, and lutein [40], as well as plant extracts [4]. The action of a thermal agent on a chemical can result in structural changes and decomposition, as well as the breaking of chemical bonds due to the energy supplied and the formation of free radicals that give the sample its paramagnetic properties [7]. The literature emphasises the influence of the oxidation and thermal degradation processes of polyphenolic compounds, including phenolic acids [42], and anthocyanins [43] on the formation of their antioxidant properties. The research results indicate that the temperature may change the mechanism of action of plant extracts with antioxidant potential [3]. In the present study, the effect of UVA radiation and a temperature of 50 °C on the free radical scavenging activity of extracts from *H. rhamnoides* and *V. oxycoccos* was investigated. The obtained results indicate the conditions under which the tested extract should be stored. It was predicted that UVA radiation could negatively affect the antioxidant properties of the extracts. Spectroscopic studies have shown that the EPR line parameters of extract samples exposed to UVA radiation vary with the duration of UVA exposure. The EPR method showed that UVA radiation decreases the antioxidant capacity of both sea buckthorn and cranberry extracts after the prolonged exposure of the extracts to this radiation. The effect of UVA radiation on the lower quenching of DPPH free radicals by the tested extracts results in an increase in the minimum amplitude value (A/A_DPPH_) of the EPR spectra of DPPH. This effect was not observed for shorter exposure times (30 min) to UVA radiation of the extracts. Considering the results for longer exposure times to UVA radiation of the tested extracts, it should be concluded that the sea buckthorn and cranberry extracts should be protected against UVA radiation during storage. It is worth noting that though changes under the influence of UVA radiation were observed, the tested extracts were still characterised by the ability to quench free radicals. For UVA-treated extracts, the EPR spectra of DPPH free radicals decrease due to the interaction with sea buckthorn and cranberry extracts, confirming the preservation of their antioxidant properties after UVA exposure. Greater antioxidant properties were found in sea buckthorn fruit extract.

Studies using the EPR method have shown that, irrespective of the storage time of the extracts at an elevated temperature of 50 °C, sea buckthorn and cranberry extracts continue to quench DPPH free radicals, resulting in a reduction in the magnitude of the EPR spectra. Spectroscopic studies have shown that the EPR line parameters of the tested extracts exposed to elevated temperatures change with heating time in the case of the cranberry extract and remain constant in the case of the sea buckthorn extract. These results indicate that an elevated temperature of 50 °C significantly affects only the antioxidant properties of the cranberry extract, while the antioxidant properties of the sea buckthorn extract do not change at 50 °C. Although the antioxidant effects of sea buckthorn extract increase as a result of the increased storage temperature of the extract, it should be recommended to avoid placing this extract in a 50 °C environment, as the observed increase in DPPH free radical quenching may probably be due to the thermal generation of free radicals in the plant material. In addition, a recombination effect of thermally generated free radicals and DPPH free radicals in the cranberry extract may possibly occur under these conditions.

The results of this study indicate that *H. rhamnoides* and *V. oxycoccos* extracts can be used in cosmetics to complement synthetic antioxidants. This is confirmed by the results of our own research [44], in which it was shown that European cranberry fruit glycolic extract can be used as a natural antioxidant, effectively protecting cosmetic preparations, which, moreover, provides good care effects. According to the data presented in this study, the 5% *V. oxycoccos* extract significantly reduces the adverse changes occurring during the storage of the tested O/W emulsion. The results of the peroxide number determination for the fresh emulsion and for the emulsion subjected to accelerated ageing (use of high temperature) showed that the antioxidant properties of the tested extract are similar to the protective effect of the synthetic antioxidant BHT. The analysis of sensory properties, including the evaluation of the consistency, spreadability, viscosity, gloss, absorption of the emulsion, and degree of skin smoothness, as well as hedonistic analysis, i.e., evaluation of the degree of acceptance of the emulsion by consumers, turned out to be positive [44]. Similar conclusions were reached by Sikora et al. [16], who in their study evaluated the possibility of using sea buckthorn fruit glycolic extract in emulsion cosmetic preparations. The 4% sea buckthorn extract, used as an ingredient of topical emulsions, has been demonstrated to reduce the adverse changes occurring during the storage of oil-in-water (O/W) emulsion at 40 °C for 6 weeks (accelerated aging testing). The evaluation of the sea buckthorn extract’s antioxidant properties was associated with the determination of the peroxide value (LOO), which characterises the extent of adverse changes occurring during the storage of products, immediately after the preparation of emulsion, and after the accelerated aging test. It is worth stressing that the protective effect depended on the concentration of the extract used. The authors have reported the temperature-dependent colour change from orange to yellow in emulsions containing 2% sea buckthorn extract during the accelerated aging test, which may indicate adverse changes associated with the thermal and oxidative degradation of carotenoid dyes and a decrease in the antioxidant potential of the extract tested. The sensory and hedonic evaluation of the emulsion based on the sea buckthorn extract demonstrated that the preparation is well absorbed, leaving a delicate protective film on the skin, which does not cause discomfort at the application site and effectively smooths the skin [16]. The sea buckthorn extract has also been examined as an ingredient of water-in-oil (W/O) emulsion for its effect on the mechanical parameters of the skin. The results show that the sea buckthorn extract applied externally significantly improves facial skin parameters, such as elasticity and hydration [15], reduces transepidermal water loss (TEWL) [24], and improves the barrier function of human skin [Hernandez], which may be associated with the content of natural antioxidants, including flavonoids, ascorbic acid, tocopherols, and carotenoids [15,24]. The results of the studies discussed above indicate that the sea buckthorn extract can be used as a natural ingredient in topical skin protective and skin care preparations.

## 4. Materials and Methods

### 4.1. Plant Material

The material for the research consisted of *Hippophaë rhamnoides* and *Vaccinium oxycoccos* fruit glycolic extracts (Naturex, Katowice, Poland) with a defined chemical composition (Table 1) determined in the authors’ previous research [19].

In the present study, extracts stored at room temperature and exposed to a temperature of 50 °C using a dry hot air thermo-circulating chamber (Memmert, Schwabach, Germany) were tested. In addition, the extract samples were irradiated from a distance of 30 cm from an ultraviolet source in the UVA range (λ = 315–380 nm) using a Medisun 250 lamp (Schulze & Bohm, Brühl, Germany). Four UVA and elevated temperature exposure times were used in the experiment: 30, 60, 90, and 120 min.

### 4.2. EPR Measurements

The antioxidant interactions of *H. rhamnoides* and *V. oxycoccos* extracts were examined by the use electron paramagnetic resonance (EPR) spectroscopy. An ethanolic solution of DPPH (2,2-difenyl-1-picrylhydrazyl) (Sigma-Aldrich, St. Louis, MO, USA) was used as the standard of free radicals. The ethanolic solution of DPPH was obtained by dissolving 5 mg of DPPH in 100 mL of ethanol. Then, the prepared solution was stirred for 60 min using a magnetic stirrer. The DPPH free radical has an unpaired electron on the nitrogen (N) atom in its structure, which gives it paramagnetic properties.

The free radical scavenging activity of the tested extracts was determined by the electron paramagnetic resonance measurements of the quenching of DPPH free radicals’ EPR spectra after the addition of the extracts to DPPH in ethyl alcohol solution. In a ratio of 1 to 10 (*v*/*v*), extracts were added to the DPPH solution.

EPR measurements were performed at room temperature with the use of an X-band (9.3 GHz) electron paramagnetic resonance spectrometer (Radiopan, Poznań, Poland) with a magnetic modulation of 100 kHz. The numerical acquisition of data was performed with the use of the Rapid Scan Unit (Jagmar, Kraków, Poland), which was linked to the spectrometer. The conditions for EPR measurements were as previously described [45]. Briefly, the total microwave power produced by the klystron was 70 mW (M_o_). The microwave frequency was directly obtained by the MCM101 recorder (Eprad, Poznań, Poland). The kinetics of the interactions of the extract samples were obtained from the EPR spectra of DPPH free radicals detected in 1 min intervals up to 15 min. The EPR spectra were recorded as the first-derivative lines. To avoid microwave saturation, the EPR spectra of DPPH free radicals were measured with a low microwave power of 2.2 mW, which corresponded to an attenuation of 15 dB. The microwave power used during the EPR measurements (M) was obtained from the following formula:

attenuation [dB] = 10 lg (M/M_o_),

where the following variables are used:

M_o_—total microwave radiation power produced by the klystron (70 mW);

M—the microwave radiation power used when measuring the EPR spectrum.

The EPR measurements and the analysis of the recorded spectra were conducted with the use of professional spectroscopic programs of Jagmar (Kraków, Poland), LabView 8.2 (National Instruments, Austin, TX, USA), and OriginPro 2015 (OriginLab, Northampton, MA, USA).

### 4.3. Analysed Parameters of EPR Spectra

**Figure 15:**
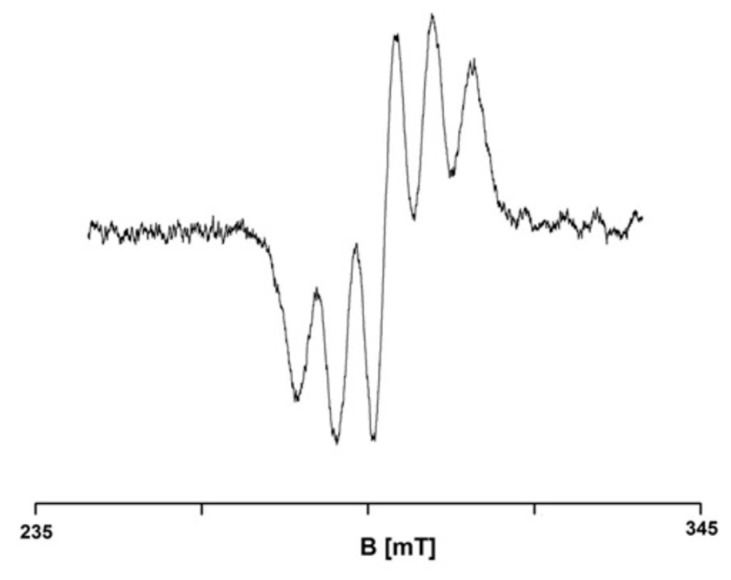
EPR spectrum of DPPH in (96%) ethyl alcohol solution. B—magnetic induction.

In the experiment, the amplitudes (A_DPPH_) of the EPR spectra of DPPH in the standard solution (Figure 15) and the amplitudes (A) of the EPR spectra of DPPH in contact with the tested plant extracts were determined. The relative amplitudes (A/A_DPPH_), whose values depend on the quenching of DPPH free radicals by substances with antioxidant properties, were analysed. For antioxidants, the values of the relative amplitudes (A/A_DPPH_) of the EPR lines are less than 1 because antioxidants quench DPPH free radicals, and the value of the amplitude (A) is smaller than the value of the amplitude (A_DPPH_). With an increase in the quenching of DPPH free radicals by stronger antioxidants, the relative amplitude value (A/A_DPPH_) of the EPR line decreases.

For the extracts tested, the kinetics of the interaction with DPPH free radicals were determined as the dependence of the relative amplitude (A/A_DPPH_) of the DPPH EPR line on the time (t) of interaction of the extract with DPPH. For antioxidants, the relative amplitude (A/A_DPPH_) of the EPR line of DPPH decreases with an increasing interaction time with DPPH free radicals and, after reaching a minimum value, does not change with a further increase in time. The magnitude of the interaction of an extract with DPPH free radicals was determined as the value of the minimum relative amplitude (A/A_DPPH_) of the DPPH EPR line. The g-factor [±0.0002] for the DPPH EPR lines was calculated from the resonance formula as follows [45]:

g = hν/μ_B_B_r_,

where the following variables are used:

h—Planck constant;

ν—microwave frequency;

μ_B_—Bohr magneton;

B_r_—induction of resonance magnetic field.

The g-factor depends on the type of free radical. Measurements were performed in triplicate. The maximum error was determined for the EPR line amplitudes. The maximum error for the relative amplitude (A/A_DPPH_) of the DPPH EPR line was ±0.05 [a.u.].

## 5. Conclusions

The studies carried out in this paper using EPR spectroscopy have provided information and expanded the existing knowledge on the interactions of plant extracts with free radicals.

The outcomes of this study indicate that *Hippophaë rhamnoides* and *Vaccinium oxycoccos* fruit glycolic extracts can be used as valuable raw materials with antioxidant properties. The decrease in the EPR spectra of DPPH as a result of the interaction of extracts with free radicals indicates the quenching of free radicals by these extracts and their antioxidant nature. EPR spectroscopic analyses indicate that the sea buckthorn extract interacts more strongly with free radicals than the cranberry extract. The *H. rhamnoides* extract causes greater quenching of the EPR signal of DPPH free radicals. It was also shown that the interaction kinetics of extract mixtures containing sea buckthorn and cranberry extracts in volume ratios of 2:1 and 1:2 are similar, while the mixture of these extracts in a volume ratio of 2:1 quenches free radicals and EPR spectra more strongly. Considering the antioxidant effects, it is therefore possible to propose the use of the mixture of glycolic extracts with a higher content of sea buckthorn extract than cranberry extract in cosmetic products.

The results obtained indicate the conditions under which the tested extracts should be stored. It was shown that a longer duration of UVA radiation exposure decreases the free radical scavenging capacity of both the sea buckthorn extract and cranberry extract. Sea buckthorn and cranberry extracts should be protected from UVA radiation during storage. Furthermore, it was shown that storing the sea buckthorn extract at an elevated temperature of 50 °C does not affect the extract’s interactions with free radicals. In contrast, an elevated temperature of 50 °C affects the free radical interactions of cranberry extract, so the extract should not be exposed to this temperature during storage.

The research performed confirmed the usefulness of EPR spectroscopy for assessing the interaction of plant extracts with free radicals. The results of the present study and the experimental methodology may be useful in the search for the optimal storage conditions for plant extracts used as cosmetic and pharmaceutical raw materials.

## Figures and Tables

**Table 1:** Total content of polyphenols and selected groups of polyphenolic compounds of sea buckthorn and European cranberry fruit glycolic extracts.

Extract	Content (mg/100 mL of Extract)				
	Total Polyphenols Content (a)	Flavonols (b)	Hydroxybenzoic Acids (c)	Hydroxycinnamic Acids (d)	Proanthocyanidins (a)
*H. rhamnoides*	60.85	17.00	3.91	0.32	8.08
*V. oxycoccos*	21.39	0.54	14.11	0.38	10.08

(a) determined by spectrophotometric method; (b) determined by HPLC method and calculated as quercetin; (c) determined by HPLC method and calculated as gallic acid; (d) determined by HPLC method and calculated as chlorogenic acid.

## Data Availability

All data are presented in this manuscript.
